# CD8+ T Cells Primed by Antigenic Peptide-Pulsed B Cells or Dendritic Cells Generate Similar Anti-Tumor Response

**DOI:** 10.3390/vaccines13090953

**Published:** 2025-09-06

**Authors:** Ichwaku Rastogi, Wanyi Guo, Jena E. Moseman, Douglas G. McNeel

**Affiliations:** Carbone Cancer Center, University of Wisconsin, Madison, WI 53705, USA; wguo75@wisc.edu (W.G.); jemoseman@wisc.edu (J.E.M.)

**Keywords:** cellular vaccines, APC-based therapies, DC vaccines, B cell vaccines, adoptive cell therapies

## Abstract

**Background:** Peptide-loaded antigen-presenting cell (APC)-based vaccines have been under investigation as a therapeutic approach for treating cancer. However, in general they have demonstrated limited efficacy in clinical trials. Dendritic cells (DCs) have been the primary choice for APC-based vaccines given their ability to cross-present antigens. B cells have been less studied as APCs for vaccines. Here we compare the phenotype and anti-tumor activity of activated T cells that result from peptide-specific priming using either B cells or DCs. **Methods:** B cells and DCs were isolated from C57Bl/6 mice, and either treated or not treated with lipopolysaccharide (LPS) for maturation, and then either loaded or not loaded with SIINFEKL peptide to prime CD8+ T cells from OT-1 mice. Activated T cells were then analyzed for their phenotype and anti-tumor efficacy. **Results:** We report that both immature B cells and immature DCs were similarly capable of activating antigen-specific CD8+ T cells. However, LPS-matured DCs generated a stronger CD8+ T cell activation profile *in vitro* compared to LPS-matured B cells. Immature B cells, mature DCs and immature DCs all generated a similar anti-tumor response upon adoptive transfer of primed CD8+ T cells to tumor-bearing mice. **Conclusions:** Collectively, our data suggests that B cells and DCs are each capable of priming CD8+ T cells and generating anti-tumor responses. Given that B cells are relatively easier to culture and expand compared to DCs, our study suggests that, following further validation, B cells could be further investigated as APCs for peptide-based human cancer vaccines.

## 1. Introduction

Antigen presenting cell (APC)-based vaccines can be a direct way of eliciting an antigen-specific anti-tumor response, by activating tumor-specific CD8+ T cells. The conceptual idea behind APC vaccines is to deliver APCs loaded with tumor-associated antigen(s) such that they result in priming of T cells for anti-tumor inflammatory and cytotoxic responses [1]. The tumor-associated antigen could be in the form of a protein, nucleic acid, bacterial/viral vector, or peptide. Among the professional APC subsets, dendritic cells (DCs) have been of greater interest for the development of these cell-based vaccines [2,3,4,5], as they have been demonstrated as the primary cross-presenter of the antigenic protein produced by tumors to activate CD8+ T cells [6]. Moreover, they have been accredited with most of the cross-priming and cross-dressing that occur in humans to elicit antigen specific immune responses [7,8].

Currently, there is only one FDA-approved APC-based vaccine, Sipuleucel-T. This was approved for the treatment of metastatic castration-resistant prostate cancer [9]. It comprises patient blood cells that are enriched for APCs, and loaded ex vivo with an antigenic protein comprising a tissue-specific antigen fused to GM-CSF, which is being used in part to help mature APCs [10]. Other DC-based vaccines that are either currently under development or have failed in clinical trials include the use of *in vitro* generated monocyte-derived DCs, exosomes derived from DCs, *in vivo* DC-targeted vaccines, DCs generated *in vitro* from CD34+ hematopoietic precursors, naturally circulating blood DCs, allogeneic plasmacytoid DC lines, and plasmacytoid DC-derived exosomes [11]. A large phase III trial tested mature monocyte-derived dendritic cells, co-electroporated with amplified tumor RNA and CD40L RNA (Rocapuldencel-T) as an intervention in combination with standard of care (SOC) for treatment of patients with metastatic renal cell carcinoma (NCT01582672). In this trial of 462 patients, while immune responses were detected in 70% of the 307 patients treated with Rocapuldencel-T, there was no improvement in overall survival or progression-free survival [12].

Other APC subsets, such as monocytes and macrophages, have been investigated as cellular therapies, but have not been extensively evaluated as vaccine strategies [13]. However, a few studies have suggested that B cells may be effective as a means of delivering a vaccine antigen. In one approach, allogeneic B cells from healthy donors were fused with autologous tumor cells for the treatment of renal cell carcinoma [14]. This study reported two partial and two complete responses out of 11 patients [14]. In another clinical trial, allogeneic B cells from healthy donors were fused with autologous tumor cells and used to treat patients with metastatic melanoma. The trial reported one complete response, one partial response, and five patients with a stable disease out of 16 patients [15]. With only minor side effects reported, these trials demonstrated that B cell-based approaches are safe and can potentially generate effective anti-tumor responses.

The failures of most DC-based vaccine approaches, coupled with encouraging results seen in small B cell-based vaccine approaches, have led to a renewed interest in the use of B cells to deliver vaccine antigens [16,17,18,19,20,21]. It has also been reported that, unlike DCs, B cell-based vaccines can be resistant to immune suppression by cytokines like IL-10, TGF-β, and VEGF [13,22]. Shimabukuro-Vornhagen et al. demonstrated that the IL-10, TGF-β or VEGF treatment of CD40-activated B cells did not inhibit their APC function, proliferation, migration or ability to stimulate CD4+ and CD8+ T cells [22]. Moreover, the use of B cells as APC vaccines can be relatively simple and economical, as a high number of circulating B cells can be purified from blood and expanded ex vivo for use as APCs [23,24]. Consequently, B cells could provide a more feasible and effective alternative to DC-based vaccines. This is particularly relevant in recent years as there has been an effort to identify novel epitopes generated by tumor-specific mutations, and use peptides specific for these mutation-associated neoantigens as vaccines [25].

In this manuscript, we sought to directly compare the ability of B cells and DCs in their mature or immature state to prime CD8+ T cells. We used a peptide-loading approach to enable a side-by-side comparison of DC and B cells, using the ovalbumin-derived SIINFEKL epitope peptide as a model antigen. Lipopolysaccharide (LPS) was chosen as the agent for the maturation of B cells and DCs, since it has been shown to be a potent activator that primes APCs for an effective immune response through TLR4 signaling, and results in the upregulation of costimulatory molecules, and enhances the antigen presentation capacity of APCs [26,27]. We further analyzed known B cell activation agents (BAFF, anti-CD40, and CD40L) for their effects on the anti-tumor function of T cells primed by differently activated B cells. We evaluated peptide-loaded APCs, as this approach eliminated the differences in the capabilities of antigen uptake and processing by different APC subsets, rather tested them directly for their ability to activate CD8+ T cells through epitope-specific priming. We also believe this approach is most directly relevant to current efforts seeking to use peptide vaccines to generate CD8+ T cells specific for mutation-associated tumor neoantigens. The CD8+ T cells primed by B cells or DCs were then evaluated for their activation and exhaustion profile, cytotoxic function, and anti-tumor function. Given the possibility that multiple differences in co-stimulatory molecules and cytokines produced by different APC subsets could affect CD8+ T cell function, our studies here focused primarily on the phenotypic differences in CD8+ T cells primed by these peptide-loaded APC subsets. 

## 2. Materials and Methods

### 2.1. Cell Lines and Mouse Models

E.G7-OVA (derivative of EL4, with constitutive expression of ovalbumin) cell line was purchased from ATCC (Manassas, VA, USA, Cat. # CRL-2113) and maintained according to ATCC-recommended guidelines.

C57Bl/6 mice (stock no. 000664) and OT1 mice (stock no. 003831) were obtained from the Jackson laboratory (Bar Harbor, ME, USA) and were housed and monitored by the Wisconsin Institute of Medical Research (Madison, WI, USA) vivarium facility. All mice were maintained under aseptic conditions and all experiments were conducted under an IACUC-approved protocol.

### 2.2. Materials

#### 2.2.1. Antibodies

*Flow Cytometry antibodies:* Anti-mouse CD25-PE (Cat. #553866), CD28-PE-CF594 (Cat. #562765), CD62L-BV650 (Cat. #564108), CD27-BUV805 (Cat. #BDB741959), CD8-BV786 (Cat. #563332), CD19-FITC (Cat. #553785), CD11c-FITC (Cat. #553801), PD1-PE-CF594 (Cat. #562523), LAG3-BV711 (Cat. #563179), CD244-BUV395 (Cat. #744290), CD160-PerCp-Cy5.5 (Cat. #562218), CD107a-BV421 (Cat. #564347), CD278 (ICOS)-BV480 (Cat. #746539), TNF-α-PE-Cy7 (Cat. #557644), IL-10-BV510 (Cat. #563277), and EOMES-BUV395 (Cat. #567171) were purchased from BD Biosciences (Franklin Lakes, NJ, USA). Anti-mouse CD40L-PE-Cy7 (Cat. #157008), OX40-APC (Cat. #119414), CTLA-4-PE-Cy7 (Cat. #106314), VISTA-PerCp-Cy5.5 (Cat. #150210), and TIGIT-PE-Cy7 (Cat. #142108) were purchased from BioLegend (San Diego, CA, USA). Anti-mouse CD44-PerCp-Cy5.5 (Cat. #560570), CD69-BV510 (Cat. #563030), FAS-L-PE (Cat. #555293), FAS-R-BV711 (Cat. #740716), Perforin-FITC (Cat. #11-9392-82), IL-4-BV421 (Cat. #566288), Ki67-BV711 (Cat. #563755), CD19-BUV805 (Cat. #749027), and CD11c-BUV805 (Cat. #749038) were purchased from Fisher Scientific (Waltham, MA, USA). Anti-mouse CD137 (4-1BB)-PerCp-eF710 (Cat. # 46-1371-82) was purchased from Life Technologies (Carlsbad, CA, USA). Ghost dye 780 (Live/dead stain) (Cat. # 13-0865-T500) was purchased from Tonbo Biosciences (San Diego, CA, USA).

*ELISA antibodies:* Purified anti-mouse IFN-γ (Cat.# 551216) and biotinylated anti-mouse IFN-γ (Cat.# 554410) were purchased from Thermo Fisher Scientific (Waltham, MA, USA). Avidin-HRP (Cat.# 170-6528) was purchased from Bio-Rad laboratories (Hercules, CA, USA).

#### 2.2.2. Reagents

Recombinant mouse GM-CSF (Cat.# 576304) and recombinant mouse BAFF (Cat.# 591202) were purchased from BioLegend (San Diego, CA, USA). Recombinant mouse IL-4 (Cat.# 21-8041-U0020) was purchased from Tonbo Biosciences (San Diego, CA, USA). RPMI-1640 (Cat.# 10-040-cv) and penicillin/streptomycin solution (Cat.# 15140122) were purchased from Thermo Fisher Scientific (Waltham, MA, USA). BenchMark FBS (Cat.# 100-106 500mL) was purchased from Gemini Bio (Sacramento, CA, USA), and TMB-substrate (Cat.# 50-76-00) was purchased from Sera Care Life Sciences (Milford, MA, USA). Recombinant mouse CD40L (Cat.# 8230-CL-050/CF) was purchased from R&D systems (Minneapolis, MN, USA). LPS (Cat.# L4516-1 mg) was purchased from Sigma Aldrich (St. Louis, MO, USA), and anti-mouse CD40 (Cat.# 553721) was purchased from BD Biosciences (Franklin Lakes, NJ, USA).

#### 2.2.3. Peptide

Peptide for the H-2Kb-restricted epitope from chicken ovalbumin (SIINFEKL) was synthesized, and the purity and identity were confirmed by mass spectrometry and gas chromatography (LifeTein, LLC., Hillsborough, NJ, USA). Peptides were reconstituted in DMSO (2 mg/mL) and stored at −80 °C until use.

### 2.3. Methods

#### 2.3.1. B Cell, DC, and T Cell Isolations

Mouse spleens were acquired at necropsy and processed to single cell suspension following red blood cell lysis [15]. B cells were isolated using a negative selection kit (Cat.# 12210-110) from Akadeum technologies (Ann Arbor, MI, USA) following the manufacturer’s protocol. B16/Flt3-L cell line was implanted in C57Bl/6 mice for the generation of primary DCs *in vivo*, as previously described [28]. DCs and CD8+ T cells were isolated by negative selection enrichment (Cat. # 19763 or Cat. # 19853) from StemCell technologies (Vancouver, BC, Canada) following the manufacturer’s protocol.

#### 2.3.2. *In Vitro* Assay

Purified B cells or DCs were plated at 2.5 × 10^5^ cells per well in a 96-well plate. Cells were cultured in mouse media (RPMI-1640 + 10% FBS + 1% penicillin/streptomycin) for all incubations. For maturation, B cells and DCs were treated with LPS (Sigma Aldrich, St. Louis, MO, USA, Cat. # L4516-1mg) at 1 µg/mL or 10 µg/mL, respectively, for 24 h. Non-LPS treated cells were used as immature B cells and DCs. Following LPS treatment, cells were washed twice with PBS and then SIINFEKL peptide was added to the culture at 1 µg/mL (or no peptide added as negative control). After four hours of incubation with peptide (or negative control), cells were washed twice with PBS and isolated CD8+ T cells (APC:T cell; 1:1) were added to the culture. Similar conditions were previously demonstrated to result in >80% of APCs being loaded with peptide [29]. At this point, GM-CSF (25 ng/mL) and IL-4 (20 ng/mL) were also added to the culture, if indicated. After 48 h of culture, CD8+ T cells were analyzed by cell surface marker and intracellular protein expression using flow cytometry, IFN-γ secretion by ELISA [20], or used for adoptive transfer to study their anti-tumor effects. For evaluating proliferation, CD8+ T cells were labeled with CFSE (BioLegend, San Diego, CA, USA, Cat. #423801) before they were added to the culture. Loss of CFSE was quantified using flow cytometry after 48 h. For flow cytometry analysis, the negative control groups (non-activated live CD8+ T cells) were used to define the gates.

#### 2.3.3. Tumor Study

C57Bl/6 male mice were implanted with E.G7-OVA cells (lymphoma cell line that expresses ovalbumin); 1 × 10^6^ cells were injected per mouse subcutaneously in the left flank region. After eight days, tumor volumes were measured, and mice were randomized to different treatment groups. Antigen-primed CD8+ T cells were collected (re-purified) after 48 h of *in vitro* culture (described above), and 1 × 10^6^ cells were intraperitoneally adoptively transferred per mouse on day 9 [30]. Tumor volumes were measured and recorded every second or third day. Mice were compassionately euthanized when tumor volumes reached 2 cm^3^.

#### 2.3.4. Statistical Analysis

All data presented are representative of at least two similar experiments/assays. The *in vivo* tumor treatment study was performed with four (negative controls) or six (experimental groups) mice per treatment group. Data are expressed as mean ± standard deviation for all *in vitro* analysis and mean ± standard error for tumor volume curves. A two-way analysis of variance (ANOVA) was used to calculate statistical significance for all data presented that had more than two groups for comparison. Standard *t*-test was used for experiments that had only two experimental groups. Survival analysis was conducted using a Mantel-Cox log-rank test. *p* < 0.05 was considered statistically significant.

## 3. Results

### 3.1. Epitope-Specific Priming of CD8+ T Cells by B Cells and DCs Resulted in Different Activation Marker Expression Profiles

**Figure 1:**
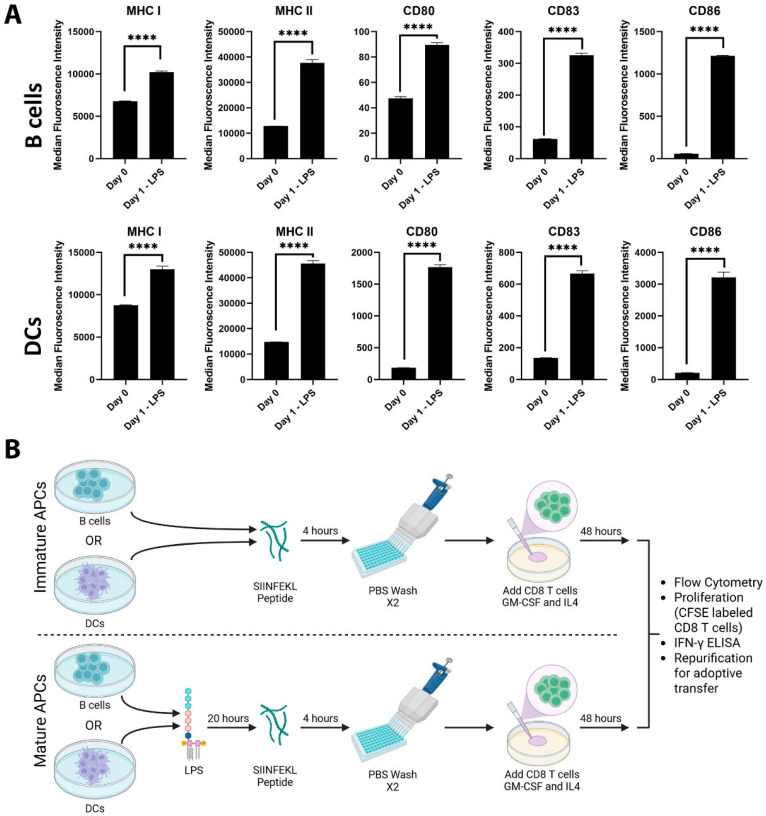
LPS treatment results in the activation of B cells and DCs. B cells and DCs isolated from the spleens of C57Bl/6 mice were either LPS treated or not. After 24 h of incubation, cells were washed and stained for flow cytometry. (**A**) Expressions of MHC I, MHC II, CD80, CD83, and CD86 were measured, asterisks **** indicate *p* < 0.0001. (**B**) Schematic for experimental set up used to evaluate T cells after priming by immature or mature APCs.

To evaluate differences in CD8+ T cell activation when primed by peptide loaded-B cells or DCs, we isolated B cells from the spleens of wild-type C56Bl/6 mice and DCs from the spleens of C57Bl/6 mice that were implanted with Flt3L-secreting B16 tumors. Mature APCs were then generated by the LPS treatment of B cells and DCs for 24 h, and non-LPS treated APCs were used as immature forms of B cells and DCs. LPS treatment resulted in the increased expression of MHC I, MHC II, CD80, CD83, and CD86 on both B cells and DCs (Figure 1A). We also evaluated the inclusion of GM-CSF and IL-4 cytokines, as it has been previously reported by us and others that the APC function of B cells and DCs could be augmented in their presence [20,31,32,33,34], and B cells have increased survival and activation in the presence of GM-CSF and IL-4 [20]. These mature or immature APC subsets were then loaded with peptide directly and evaluated for their ability to activate antigen-specific CD8+ T cells, as in Figure 1B.

**Figure 2:**
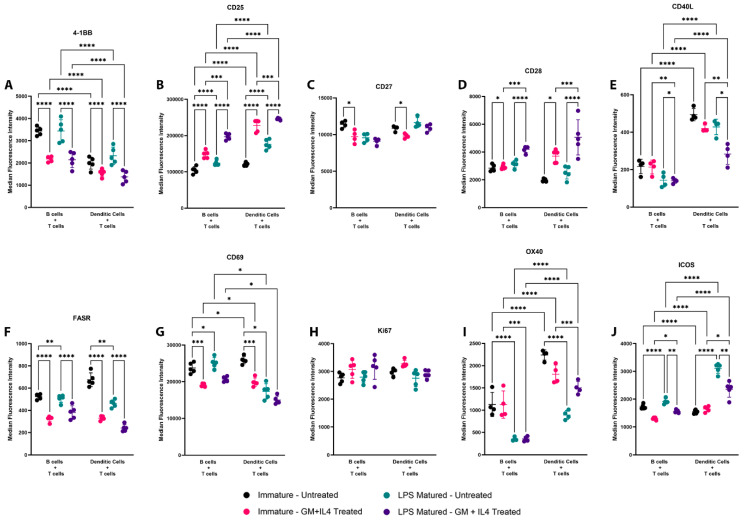
Epitope-specific priming of CD8+ T cells by B cells and DCs resulted in different activation marker expression profiles. B cells and DCs isolated from the C57Bl/6 spleen were either LPS treated or not and then loaded with SIINFEKL peptide. After 48 h of incubation with CD8+ T cells in the presence or absence of GM-CSF and IL-4, flow cytometry was performed to measure the expression of activation markers (**A**) 4-1BB, (**B**) CD25, (**C**) CD27, (**D**) CD28, (**E**) CD40L, (**F**) FASR, (**G**) CD69, (**H**) Ki67, (**I**) OX40, and (**J**) ICOS. Median fluorescence intensities (MFI) were plotted with mean and standard deviation. The percentage of CD8+ T cells positive for the expression of the respective cell surface markers are shown in Figure S2. Each treatment group was tested with five biological replicates and each data point on the plot represents a biological replicate. Asterisks * indicate *p* < 0.05, ** indicates *p* < 0.01, *** indicates *p* < 0.001, and **** indicates *p* < 0.0001. Results are representative of at least two similar, independent experiments.

First, we evaluated the expression of cell surface activation markers by median fluorescence intensity (MFI) on CD8+ T cells when primed by mature and immature B cells/DCs. We observed that priming by B cells resulted in the increased expression of 4-1BB (Figure 2A) and priming by DCs resulted in the increased expression of CD25 (Figure 2B), CD40L (Figure 2E), OX40 (Figure 2I), and ICOS (Figure 2J) on CD8+ T cells. Interestingly, the expression of CD69 (Figure 2G) was increased when CD8+ T cells were primed by immature DCs when compared to priming by immature B cells and when CD8+ T cells were primed by mature B cells in comparison to priming by mature DCs. The expression of CD27 (Figure 2C), CD28 (Figure 2D), FASR (Figure 2F), and Ki67 (Figure 2H) were not significantly different on CD8+ T cells when primed by either B cells or DCs. Following priming in the presence of GM-CSF and IL-4, we observed the decreased expression of 4-1BB, CD27 (immature APC groups only), CD40L (LPS-matured APC groups only), FASR, CD69, and ICOS (LPS-matured APC groups only), whereas there was an increased expression of CD25 and CD28. No significant changes were observed in the expression of Ki67 and OX40 on primed CD8+ T cells in terms of treatment with GM-CSF and IL-4. Following LPS maturation, we observed an increased expression of CD25 and CD28, and a decreased expression of CD40L, FASR, and CD69 (DC groups only). No changes were observed in the expression of 4-1BB, CD27, and Ki67 on CD8+ T cells when primed by either immature or LPS-matured APCs. The percentages of CD8+ T cells expressing these markers showed similar trends (Figure S1). Overall, we demonstrated that both B cells and DCs resulted in the activation of CD8+ T cells; however, the expression profile of these CD8+ T cells was slightly different among the treatment groups, and also different if these cells were LPS-matured and/or treated with GM-CSF and IL-4.

### 3.2. B Cells and DCs Both Generated Effector Memory CD8+ T Cells upon Stimulation and Resulted in CD8+ T Cell Proliferation

**Figure 3:**
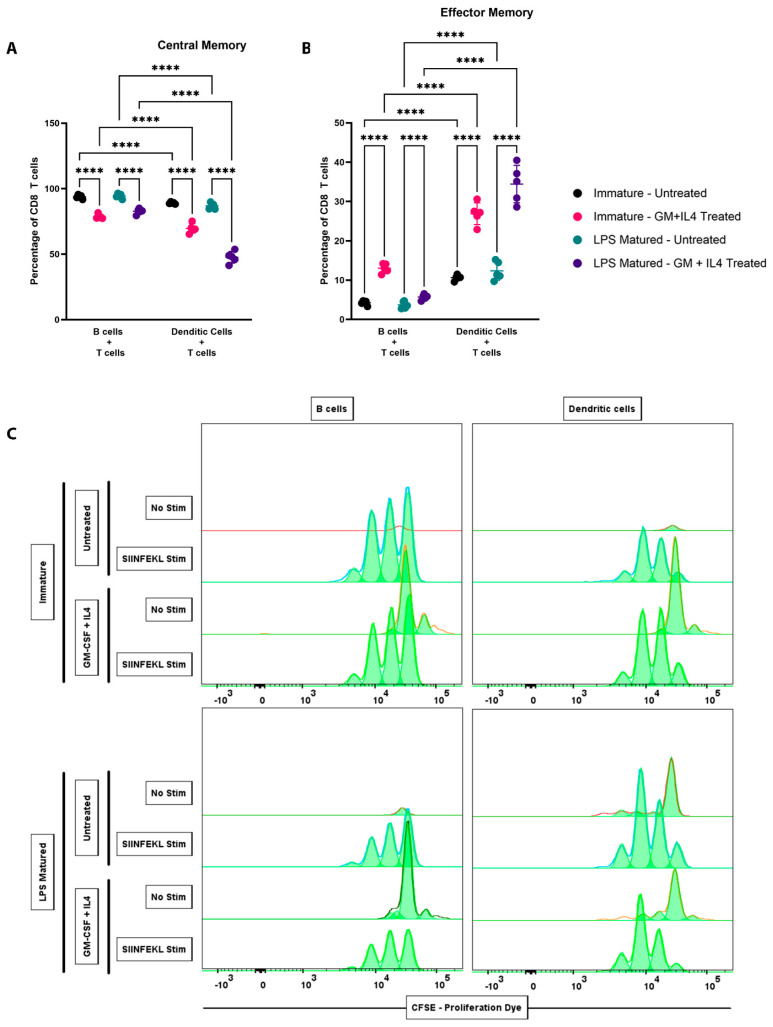
B cells and DCs both generated effector memory CD8+ T cells upon stimulation and resulted in CD8+ T cell proliferation. B cells and DCs isolated from C57Bl/6 spleen were either LPS treated or not and then loaded with SIINFEKL peptide. CD8+ T cells were labeled with CFSE and co-cultured with APCs in the presence or absence of GM-CSF and IL-4 for 48 h. Flow cytometry was performed to identify (**A**) central memory CD8+ T cells (CD44+ CD62L+) and (**B**) effector memory CD8+ T cells (CD44+ CD62L−). Each data point represents a biological replicate, the graph plotted shows the mean and standard deviation for each treatment group. (C) Representative proliferation histogram plots of CD8+ T cells as measured by loss in CFSE. Asterisks **** indicate *p* < 0.0001. Results presented are from one experiment and are representative of at least two similar, independent experiments.

As we observed different activation characteristics upon priming by B cells and DCs, we next wished to evaluate the memory phenotypes of CD8+ T cells activated by peptide-loaded B cells or DCs. CD8+ T cells primed by DCs resulted in a modest decrease in central memory (CD44+ CD62L+), and a modest increase in effector memory (CD44+ CD62L−) cells (Figure 3A,B). The inclusion of GM-CSF and IL-4 resulted in an increased percentage of effector memory cells, and a corresponding decrease in the central memory populations, when primed by either B cells or DCs (Figure 3A,B). We then evaluated the ability of peptide-loaded B cells and DCs to induce CD8+ T cell proliferation. This was achieved by tracking the loss of CFSE on the CD8+ T cells when primed by B cells or DCs. We found that both B cells and DCs were able to induce CD8+ T cell proliferation similarly, and that LPS-maturation or treatment with GM-CSF and IL-4 did not have any effect on proliferation (Figure 3C). Overall, these data showed that T cells primed by B cells and DCs were able to proliferate upon activation, and stimulation with DCs (and GM-CSF and IL-4) resulted in higher percentages of effector memory phenotype.

### 3.3. Epitope-Specific Priming of CD8+ T Cells by B Cells and DCs Resulted in Different Checkpoint- and Exhaustion-Related Marker Expression Profiles

**Figure 4:**
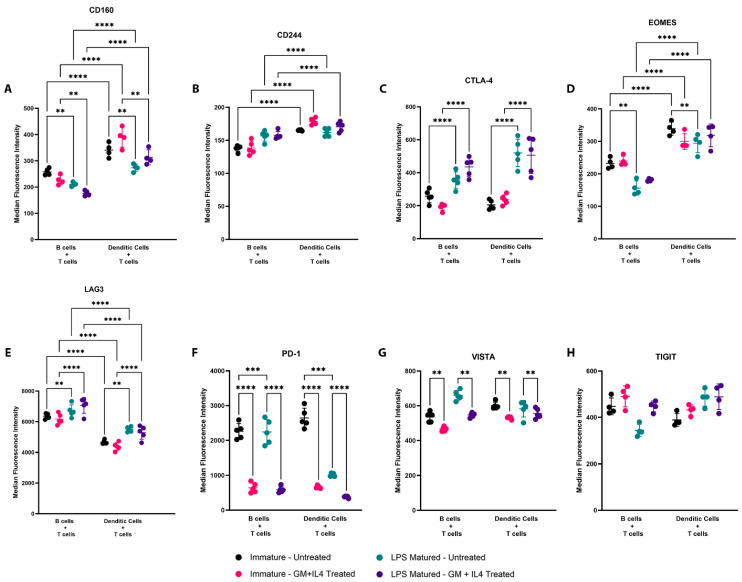
Epitope-specific priming of CD8+ T cells by B cells and DCs resulted in different checkpoint- and exhaustion-related marker expression profiles. *In vitro* assay was set up as in Figure 1B. Flow cytometry was performed to measure the expression of checkpoint and exhaustion markers (**A**) CD160, (**B**) CD244, (**C**) CTLA-4, (**D**) EOMES, (**E**) LAG3, (**F**) PD1, (**G**) VISTA, and (**H**) TIGIT. Median fluorescence intensities (MFI) were plotted for each of these markers with their mean and standard deviation. The percentage of CD8+ T cells positive for expression of the respective cell surface markers were also plotted (Figure S3). Each data point represents a biological replicate, with mean and standard deviation from five replicates. Asterisks ** indicates *p* < 0.01, *** indicates *p* < 0.001, and **** indicates *p* < 0.0001. Results are from one experiment and are representative of at least two similar, independent experiments.

We next evaluated the expression of exhaustion- and checkpoint-related markers on CD8+ T cells primed by B cells or DCs. We observed that the expression of CD160 (Figure 4A), CD244 (Figure 4B), and EOMES (Figure 4D) was increased on CD8+ T cells when primed by DCs, whereas the expression of LAG3 (Figure 4E) was increased on CD8+ T cells primed by B cells. However, expressions of CTLA-4 (Figure 4C), PD-1 (Figure 4F), VISTA (Figure 4G), and TIGIT (Figure 4H) on CD8+ T cells were similar when primed by either B cells or DCs. Following LPS maturation, we observed an increased expression of CTLA-4 and LAG-3 on CD8+ T cells, and a decreased expression of CD160, EOMES, and PD-1. Interestingly, the expression of PD-1 and VISTA was reduced when CD8+ T cells were activated in the presence of GM-CSF and IL-4, whereas the expression of other markers tested here did not significantly change with this treatment. The percentages of CD8+ T cells expressing each of these markers following the different activation methods showed similar trends (Figure S2). Overall, we conclude that the activation of CD8 T cells by B cells or DCs resulted in differences in the expression of checkpoint/exhaustion related markers, notably differences in CD160 and LAG-3. T cells activated in the presence of GM-CSF and IL-4 (or by LPS-matured DC) had a markedly decreased expression of PD-1.

### 3.4. Priming by B Cells and DCs Resulted in Differences in Expression of Cytotoxicity-Related Markers, and IL-4 and IL-10, on CD8+ T Cells

**Figure 5:**
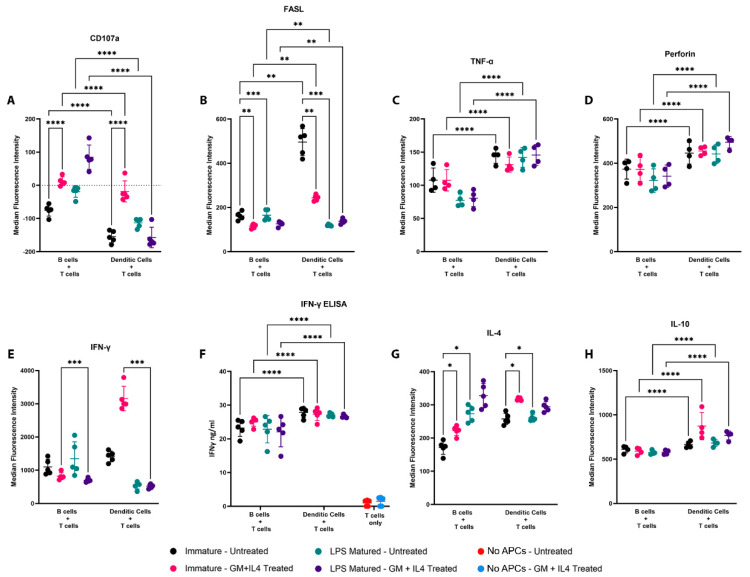
Priming by B cells and DCs resulted in differences in the expression of cytotoxicity-related markers, and IL-4 and IL-10, on CD8+ T cells. *In vitro* assay was set up as in Figure 1B. Flow cytometry was performed to measure the expression of surface and intra-cellular proteins associated with cytotoxicity. Expression levels of (**A**) CD107a, (**B**) FASL, and intracellular expression levels of (**C**) TNF-α, (**D**) Perforin, (**E**) IFN-γ, (**G**) IL-4, and (**H**) IL-10 were also quantified. Median fluorescence intensities (MFI) were plotted with mean and standard deviations. The percentage of CD8+ T cells positive for expression of the respective cell surface markers were also determined (Figure S4). Each data point represents a biological replicate, with mean and standard deviation from five replicates. (**F**) Following 48 h of co-culture, levels of secreted IFN-γ from cell culture supernatants were also quantified using ELISA. Asterisks * indicates *p* < 0.05, ** indicates *p* < 0.01, *** indicates *p* < 0.001, and **** indicates *p* < 0.0001. Results are from one experiment, with samples assessed using five biological replicates.

We next wished to characterize the CD8+ T cells based on the expression of cytotoxicity-related markers following activation by B cells or DC. As shown in Figure 5, the expression (MFI) of CD107a (Figure 5A) was increased when CD8+ T cells were primed by B cells, whereas the expression of FASL (Figure 5B), TNF-α (Figure 5C), Perforin (Figure 5D), and IFN-γ secretion (Figure 5F) were increased when CD8+ T cells were primed by DCs. Following the maturation of B cells, increases were observed in CD107a (Figure 5A) and IL-4 (Figure 5G), and decreases in TNFα (Figure 5C). Maturation of DCs led to a decreased expression of FASL (Figure 5B) and IFN-γ (Figure 5E), following CD8+ T-cell activation. Following treatment with GM-CSF and IL-4, increases in CD107a (Figure 5A) and IFN-γ (Figure 5E) expression were observed, although the secretion of IFN-γ over 48 h was not significantly different (Figure 5F). With respect to other cytokines, we observed that CD8+ T cells expressed higher levels of IL-10 when primed by DCs (Figure 5H), and the expression of IL-4 was lowest following priming with immature B cells (Figure 5G). Expression of IL-4 was higher following activation in the presence of GM-CSF and IL-4 (Figure 5G). Similar trends were observed for the percentages of CD8+ T cells positive for the expression of these markers (Figure S3). Collectively, these findings suggested that CD8+ T cells primed by DCs resulted in a higher expression of cytotoxicity-related markers, but a lower expression of CD107a.

### 3.5. tSNE Analysis Revealed Different Phenotypes of CD8+ T Cells Resulting from Priming by B Cells and DCs

**Figure 6:**
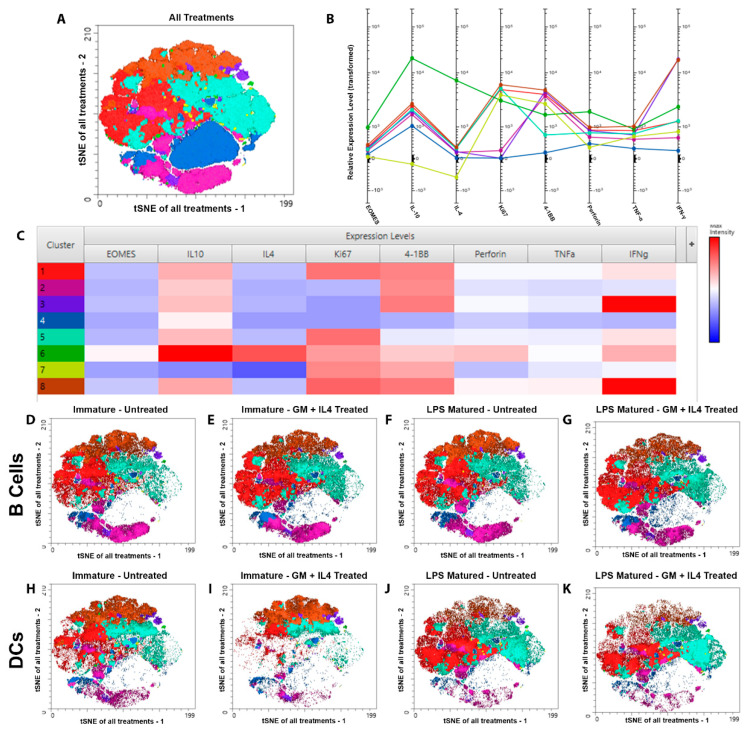
tSNE analysis revealed different phenotypes of CD8+ T cells resulting from priming by B cells and DCs. *In vitro* assay was set up as in Figure 1B. Flow cytometry was performed to measure the expression of markers used in the analysis. tSNE analysis was performed using FlowJo (version 10.10), based on expression levels of 4-1BB, EOMES, IFN-γ, IL-10, IL-4, Ki67, Perforin, and TNF-α. (**A**) A collective tSNE plot for all treatment groups, (**B**) relative expression levels of each marker with respect to the clusters identified in panel A, (**C**) heat-map defining the expression levels of each of these markers within the individual clusters, and (**D**–**K**) individual tSNE plots for each treatment group are presented. Data represented are from one experiment with five biological replicates for each treatment group.

We then characterized the functional aspects of CD8+ T cells primed by B cells/DCs by clustering based on multiple co-expressed markers that defined the effector function of CD8+ T cells. This included 4-1BB, Ki67, IFN-γ, TNF-α, Perforin, EOMES, IL-4, and IL-10. As shown in Figure 6A–C, a total of eight clusters were defined based on the expression of these proteins. This analysis demonstrated that priming by B cells and DCs resulted in significantly different CD8+ T cell phenotypes (Figure 6D–K and Figure S4A–H). Cluster 4 (negative for all markers) was dominantly present only when CD8+ T cells were co-cultured with B cells or DCs in absence of the antigen and indicated that these were non-activated naïve CD8+ T cells (Figure S4I,L). Cluster 1 (Ki67+, 4-1BB+) represented almost half of all the events recorded in each of the treatment groups except when CD8+ T cells were primed by immature DCs in the presence of GM-CSF and IL-4 (Figure S4A–H). Cluster 3 (IFN-γ+, 4-1BB+, Ki67−), cluster 6 (IL-10+, IL-4+, Perforin+, IFN-γ+), and cluster 7 (IL-10-) were represented evenly throughout all the treatment groups tested and were present in very low numbers (Figure 6D–K and Figure S4A–H). Cluster 2 (Ki67−, 4-1BB+) was prominently present when CD8+ T cells were primed by B cells, whereas cluster 5 (Ki67+, 4-1BB^low^) was over-represented when CD8+ T cells were primed by DCs (Figure 6D–K and Figure S4A–H). Interestingly, the presence of cluster 8 (IFN-γ+, Ki67+, 4-1BB+) decreased when CD8+ T cells were primed by LPS-matured DCs. Overall, this analysis revealed that the priming of CD8+ T cells with B cells or DCs resulted in different effector phenotypes.

### 3.6. CD8+ T Cells Primed by Immature B Cells, Mature DCs, and Immature DCs Generated Similar Anti-Tumor Response

**Figure 7:**
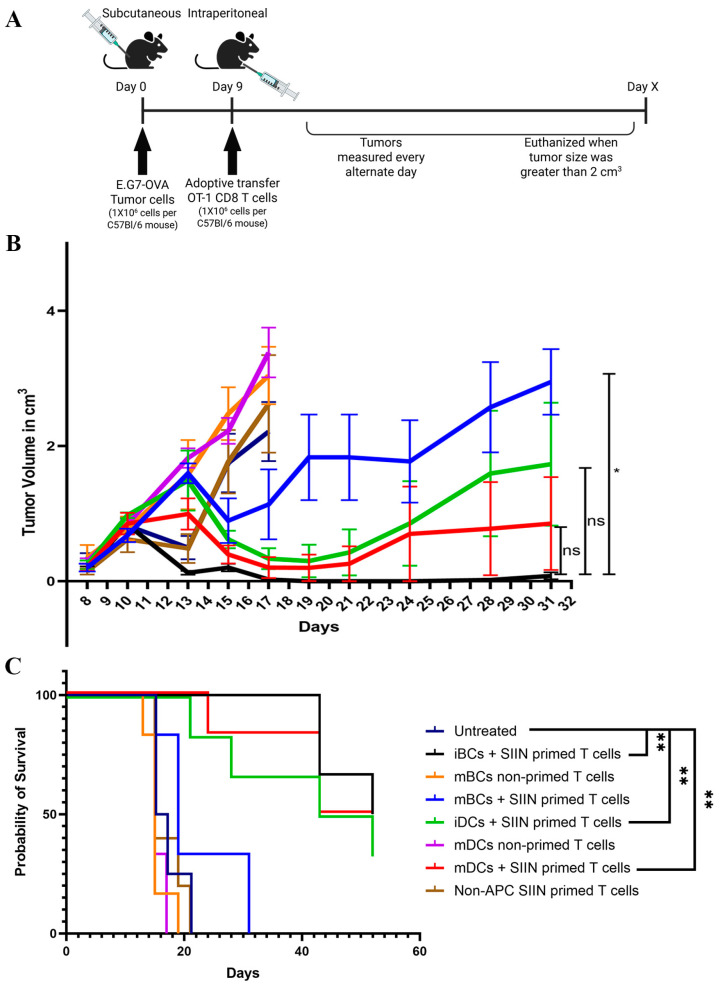
CD8+ T cells primed by immature B cells, mature DCs, and immature DCs generated similar anti-tumor response. C57Bl/6 mice were implanted with E.G7-OVA tumors subcutaneously, with six mice per treatment group (n = 4 for negative control groups not receiving transferred cells). CD8+ T cells obtained from the spleens of OT-1 mice were primed *in vitro* as in Figure 1B in the presence of GM-CSF and IL-4, and after 48 h of incubation, CD8+ T cells were re-purified using negative selection. One million re-purified CD8+ T cells were adoptively transferred to individual tumor-bearing mice on Day 9, via intra-peritoneal injection. (**A**) Schematic and timeline. (**B**) Tumor volumes (cm^3^) plotted against the day of measurement and (**C**) survival curve evaluating time to death or tumor volume ≥ 2 cm^3^, whichever occurred first. Asterisks * indicate *p* < 0.05 and ** indicates *p* < 0.01. Results presented are from one experiment, but data are representative of at least two similar, independent experiments.

Lastly, we evaluated the anti-tumor efficacy of CD8+ T cells that were primed by peptide-loaded mature or immature B cells or DCs. E.G7-OVA tumors were subcutaneously implanted in C57Bl/6 mice. CD8+ T cells isolated from OT-1 spleens were activated *in vitro* by SIINFEKL peptide-pulsed B cells or DCs (immature or LPS-matured) for 48 h in the presence of GM-CSF and IL-4. Activated CD8+ T cells were re-purified and adoptively transferred to tumor-bearing mice as illustrated in the schematic (Figure 7A). As shown in Figure 7B and in Figure S6, we found that CD8+ T cells primed by immature B cells, mature DCs, or immature DCs generated a similar anti-tumor response. However, CD8+ T cells primed by mature B cells failed to suppress tumor growth. We also observed that all mice that were treated with CD8+ T cells primed by immature B cells had no measurable tumors after 12 days of treatment (Day 20 in Figure 7B). Similarly, four out of six mice treated with CD8+ T cells primed by mature DCs were tumor free after 12 days of treatment (Day 20). Three mice from each of these groups were cured and remained tumor free, but in other mice, tumors recurred (Figure 7C). There was a significant improvement in overall survival (Figure 7C) for mice treated with CD8+ T cells primed by immature B cells, immature DCs, and LPS-matured DCs when compared to the untreated group. Since CD8+ T cells primed by LPS-matured B cells had an activated phenotype (Figure 2) but did not have substantial anti-tumor activity, we similarly evaluated other B cell activation agents (BAFF, αCD40, or CD40L). As shown in Figure S5A–C, T cells primed by B cells activated in the presence of these agents did not have improved anti-tumor efficacy compared to immature B cells. In fact, when CD8+ T cells were primed with B cells that were activated with αCD40, there was no effect on tumor growth and was similar to the untreated group, whereas there was modest treatment effect using CD8+ T cells primed with B cells activated by CD40L. Taken together, these data suggested that CD8+ T cells primed by immature B cells or DCs have similar anti-tumor functions. However, the maturation of B cells with LPS or other activation agents resulted in no improvement in, or reduced, anti-tumor efficacy.

## 4. Discussion

APCs play a pivotal role in initiating and shaping CD8+ T cell responses, particularly in the context of cancer immunotherapy. While DCs have long been considered the gold standard for T cell priming, it is known that B cells also possess APC capabilities and may offer practical advantages in clinical settings. In this study, we directly compared B cells and DCs for their capacity to prime CD8+ T cells and evaluated the functional consequences on anti-tumor immunity. We demonstrated that CD8+ T cells primed by either B cells or DCs displayed an activated phenotype with variable levels of exhaustion- and cytotoxicity-related marker expression. Moreover, CD8+ T cells primed by immature B cells or DCs, or LPS-matured DCs, generated a similar anti-tumor response. However, LPS-matured B cells failed to do the same. To our knowledge, this is the first study that directly compared both B cells and DCs for their ability to prime CD8+ T cells and evaluated the anti-tumor function of these CD8+ T cells.

We observed that when CD8+ T cells were primed in the presence of GM-CSF and IL-4, it resulted in a decreased expression of activation markers (4-1BB, CD40L, FASR, CD69, and ICOS), a decreased expression of checkpoint and exhaustion markers (PD-1 and VISTA), while increasing the percentage of effector memory CD8+ T cells. While GM-CSF is known for driving the differentiation of dendritic cells, it has also been implicated to influence T cell polarization and activation [35,36,37]. Similarly, IL-4 has been shown to promote T cell survival, proliferation, and to differentially regulate activation when introduced at the time of TCR-triggered activation [38]. Chen et al. demonstrated that, together, these two cytokines resulted in the maturation of B cells and T cells, the production of CD209+ DCs, and stimulated antigen-specific IgG responses in humanized mice [39]. These findings are in line with our observations which demonstrated that the presence of GM-CSF and IL-4 during CD8+ T cell activation resulted in more favorable CD8+ T cell effector phenotypes.

CD8+ T cells primed by immature DCs displayed an increased expression of some activation markers (CD40L, FASR, and OX40), increased expression of some checkpoint-related markers (CD160, EOMES, and PD-1), and increased expression of cytotoxicity-related markers (FASL and IFN-γ), whereas CD8+ T cells primed by LPS-matured DCs showed an increased expression of other activation markers (CD25, CD28, and ICOS), and an increased expression of other checkpoint-related markers (CTLA-4 and LAG-3) while generating an equivalent proportion of effector memory phenotype. LPS is a known activator of DCs which promotes antigen presentation. Biscari et al. demonstrated using a Trypanosoma cruzi infection mouse model that immunization with LPS-activated DCs elicited effector memory CD8+ T cells, which were characterized by an increased expression of CD25 and CD69 [26]. Although priming by LPS-matured or immature DCs resulted in different activation and exhaustion profiles of CD8+ T cells, there were no differences in their function in terms of anti-tumor activity. This could be due to the fact that both DC subsets were peptide-pulsed and did not have to process the antigen for presentation, bypassing the need for DC uptake and presentation.

When CD8+ T cells were primed by LPS-matured B cells, we observed that there was an increased expression of activation markers (CD25, CD28, CD69, and ICOS), an increased expression of exhaustion/checkpoint-related markers (CTLA-4 and LAG-3), and an increased expression of IL-4. On the other hand, CD8+ T cells primed by immature B cells showed an increased expression of other activation markers (CD27, CD40L, FASR, and OX40), an increased expression of exhaustion-related markers (CD160 and EOMES), and an increased expression of IFN-γ. LPS has been demonstrated to activate and promote the maturation of B cells that were characterized by the secretion of IL-6 [40,41]. Moreover, LPS-maturated B cells can influence T cell polarization in a dose-dependent manner, where low doses (<10 ng/mL) promoted a T helper type 2 polarization and high doses (>0.1 µg/mL) favored a T regulatory type 1 polarization [42]. Additionally, previous reports have shown that LPS-matured B cells resulted in anergic CD8+ T cells [43,44], whereas CD40L-activated B cells can trigger CD8+ T cells for cytotoxic responses [45]. These differences result from the T-dependent vs. T-independent mechanisms of the activation of B cells. Our findings similarly showed that CD8+ T cells primed by LPS-matured B cells failed to control tumor growth, whereas priming by immature or CD40L-treated B cells resulted in delayed tumor growth.

When directly compared, peptide-pulsed immature B cells and immature DCs activated CD8+ T cells and resulted in similar anti-tumor efficacy. However, each cell type presents distinct translational advantages and limitations. DC-based vaccines, especially those employing peptide-pulsed autologous DCs, have demonstrated safety and some immunogenicity in clinical settings, though durable clinical responses remain modest. Limitations of DCs include their limited expansion capacity [13], susceptibility to tumor-induced suppression [46], and poor trafficking to lymphoid tissues [47]. B cells, by contrast, are easier to expand *ex vivo*, less susceptible to certain forms of tumor-mediated immunosuppression, and more amenable to genetic engineering. Preclinical studies have shown that antigen-loaded or CD40L-activated B cells can elicit effective anti-tumor immunity, and early-phase clinical efforts are underway to explore their therapeutic use [18,19,45,48,49]. Despite this promise, peptide-pulsed B cell vaccines have yet to be thoroughly evaluated in human trials.

While our study highlights the potential of B cells as viable alternatives to DCs for T cell priming, it is important to recognize certain limitations. First, our experiments relied on peptide-pulsed APCs, which bypass the natural antigen uptake, processing, and presentation steps. Thus, there could be differences using protein- or nucleic acid-loaded APC vaccines that require antigen uptake and processing. Additionally, the use of a single antigen and tumor model limits generalizability, and it will be important for future studies to similarly evaluate peptides with different binding affinities for MHC and T cell receptors. Moreover, our studies were focused on the phenotypic differences in CD8+ T cells primed by these different APC subsets. There are likely multiple mechanisms for these differences which will need to be evaluated in future studies. Notwithstanding, our studies demonstrate that B cells may be an alternative to DC for peptide-loaded vaccine approaches, an approach that is in common use for delivering mutation-associated neoantigen vaccines. Future studies, however, should evaluate B cell- and DC-priming using whole protein antigens, tumor lysates, or nucleic acid-based antigens (e.g., mRNA, DNA) to evaluate other therapeutic vaccine approaches.

## 5. Conclusions

In summary, in this study we directly compared B cells and DCs for their capacity to prime CD8+ T cells following direct peptide loading, and evaluated the functional consequences of B cell- or DC-activated CD8+ T cells on anti-tumor immunity. This is of particular relevance because the use of peptide-based vaccines targeting mutation-associated neoantigens is being actively pursued by many groups as a therapeutic strategy. We demonstrated that CD8+ T cells primed by either B cells or DCs displayed an activated phenotype with variable levels of exhaustion- and cytotoxicity-related marker expression. Moreover, CD8+ T cells primed by immature B cells or DCs, or LPS-matured DCs, generated a similar anti-tumor response. Collectively, our data suggests that B cells and DCs are each capable of priming CD8+ T cells and generating anti-tumor responses. Given that B cells are relatively easier to culture and expand compared to DCs, our study suggests that, following further validation, B cells could be further investigated as APCs for peptide-based human cancer vaccines.

## Data Availability

The data used to support the findings of this study are included within the primary data or Supplementary Information file(s). Supporting data can be made available for research use upon reasonable request.

## Supplementary Materials

The following supporting information can be downloaded at: https://www.mdpi.com/article/10.3390/vaccines13090953/s1, Figure S1: Epitope-specific priming of CD8+ T cells by B cells and DCs resulted in different activation marker expression profiles; Figure S2: Epitope-specific priming of CD8+ T cells by B cells and DCs resulted in different checkpoint- and exhaustion-related marker expression profiles; Figure S3: Priming by B cells and DCs resulted in differences in expression of cytotoxicity-related markers, and IL-4 and IL-10, on CD8+ T cells; Figure S4: tSNE analysis revealed different phenotypes of CD8+ T cells resulting from priming by B cells and DCs; Figure S5: CD8+ T cells primed by matured B cells did not improve anti-tumor response; Figure S6: CD8+ T cells primed by immature B cells, mature DCs, and immature DCs generated similar anti-tumor response.

## References

[1] Sabado R.L., Meseck M., Bhardwaj N. (2016). Dendritic Cell Vaccines. Methods Mol. Biol..

[2] Palucka K., Banchereau J. (2013). Dendritic-Cell-Based Therapeutic Cancer Vaccines. Immunity.

[3] Reardon D.A., Mitchell D.A. (2017). The development of dendritic cell vaccine-based immunotherapies for glioblastoma. Semin. Immunopathol..

[4] Saxena M., Bhardwaj N. (2018). Re-Emergence of Dendritic Cell Vaccines for Cancer Treatment. Trends Cancer.

[5] Wculek S.K., Cueto F.J., Mujal A.M., Melero I., Krummel M.F., Sancho D. (2020). Dendritic cells in cancer immunology and immunotherapy. Nat. Rev. Immunol..

[6] Steinman R.M. (2012). Decisions About Dendritic Cells: Past, Present, and Future. Annu. Rev. Immunol..

[7] Bevan M.J. (1976). Cross-priming for a secondary cytotoxic response to minor H antigens with H-2 congenic cells which do not cross-react in the cytotoxic assay. J. Exp. Med..

[8] Joffre O.P., Segura E., Savina A., Amigorena S. (2012). Cross-presentation by dendritic cells. Nat. Rev. Immunol..

[9] Kantoff P.W., Higano C.S., Shore N.D., Berger E.R., Small E.J., Penson D.F., Redfern C.H., Ferrari A.C., Dreicer R., Sims R.B. (2010). Sipuleucel-T Immunotherapy for Castration-Resistant Prostate Cancer. N. Engl. J. Med..

[10] Anassi E., Ndefo U.A. (2011). Sipuleucel-T (provenge) injection: The first immunotherapy agent (vaccine) for hormone-refractory prostate cancer. Pharm. Ther..

[11] Sabado R.L., Balan S., Bhardwaj N. (2017). Dendritic cell-based immunotherapy. Cell Res..

[12] Figlin R.A., Tannir N.M., Uzzo R.G., Tykodi S.S., Chen D.Y., Master V., Kapoor A., Vaena D., Lowrance W.T., Bratslavsky G. (2020). Results of the ADAPT Phase 3 Study of Rocapuldencel-T in Combination with Sunitinib as First-Line Therapy in Patients with Metastatic Renal Cell Carcinoma. Clin. Cancer Res..

[13] Martin-Lluesma S., Graciotti M., Grimm A.J., Boudousquié C., Chiang C.L., Kandalaft L.E. (2020). Are dendritic cells the most appropriate therapeutic vaccine for patients with ovarian cancer?. Curr. Opin. Biotechnol..

[14] Kugler A., Seseke F., Thelen P., Kallerhoff M., Müller C., Stuhler G., Ringert R.H. (1998). Autologous and allogenic hybrid cell vaccine in patients with metastatic renal cell carcinoma. Br. J. Urol..

[15] Trefzer U., Weingart G., Chen Y., Herberth G., Adrian K., Winter H., Audring H., Guo Y., Sterry W., Walden P. (2000). Hybrid cell vaccination for cancer immune therapy: First clinical trial with metastatic melanoma. Int. J. Cancer.

[16] Rossetti R.A.M., Lorenzi N.P.C., Yokochi K., Rosa M.B.S.d.F., Benevides L., Margarido P.F.R., Baracat E.C., Carvalho J.P., Villa L.L., Lepique A.P. (2018). B lymphocytes can be activated to act as antigen presenting cells to promote anti-tumor responses. PLoS ONE.

[17] Zahm C.D., Colluru V.T., McNeel D.G. (2017). DNA vaccines for prostate cancer. Pharmacol. Ther..

[18] von Bergwelt-Baildon M.S., Vonderheide R.H., Maecker B., Hirano N., Anderson K.S., Butler M.O., Xia Z., Zeng W.Y., Wucherpfennig K.W., Nadler L.M. (2002). Human primary and memory cytotoxic T lymphocyte responses are efficiently induced by means of CD40-activated B cells as antigen-presenting cells: Potential for clinical application. Blood.

[19] Lapointe R., Bellemare-Pelletier A., Housseau F., Thibodeau J., Hwu P. (2003). CD40-stimulated B lymphocytes pulsed with tumor antigens are effective antigen-presenting cells that can generate specific T cells. Cancer Res..

[20] Rastogi I., McNeel D.G. (2023). B cells require licensing by dendritic cells to serve as primary antigen-presenting cells for plasmid DNA. OncoImmunology.

[21] Rastogi I., Jeon D., Moseman J.E., Muralidhar A., Potluri H.K., McNeel D.G. (2022). Role of B cells as antigen presenting cells. Front. Immunol..

[22] Shimabukuro-Vornhagen A., Draube A., Liebig T.M., Rothe A., Kochanek M., von Bergwelt-Baildon M.S. (2012). The immunosuppressive factors IL-10, TGF-β, and VEGF do not affect the antigen-presenting function of CD40-activated B cells. J. Exp. Clin. Cancer Res..

[23] Su K.-Y., Watanabe A., Yeh C.-H., Kelsoe G., Kuraoka M. (2016). Efficient Culture of Human Naive and Memory B Cells for Use as APCs. J. Immunol..

[24] Kondo E., Gryschok L., Klein-Gonzalez N., Rademacher S., Weihrauch M.R., Liebig T., Shimabukuro-Vornhagen A., Kochanek M., Draube A., Von Bergwelt-Baildon M.S. (2008). CD40-activated B cells can be generated in high number and purity in cancer patients: Analysis of immunogenicity and homing potential. Clin. Exp. Immunol..

[25] Katsnelson A. (2016). Mutations as munitions: Neoantigen vaccines get a closer look as cancer treatment. Nat. Med..

[26] Biscari L., Kaufman C.D., Farré C., Huhn V., Pacini M.F., Balbi C.B., Gómez K.A., Pérez A.R., Alloatti A. (2022). Immunization With Lipopolysaccharide-Activated Dendritic Cells Generates a Specific CD8+ T Cell Response That Confers Partial Protection Against Infection With Trypanosoma cruzi. Front. Cell Infect. Microbiol..

[27] Venkataraman C., Shankar G., Sen G., Bondada S. (1999). Bacterial lipopolysaccharide induced B cell activation is mediated via a phosphatidylinositol 3-kinase dependent signaling pathway. Immunol. Lett..

[28] Kapadia D., Sadikovic A., Vanloubbeeck Y., Brockstedt D., Fong L., Kremer E.J. (2011). Interplay between CD8α+ Dendritic Cells and Monocytes in Response to Listeria monocytogenes Infection Attenuates T Cell Responses. PLoS ONE.

[29] Rastogi I., Mannone J.A., Gibadullin R., Moseman J.E., Sidney J., Sette A., McNeel D.G., Gellman S.H. (2025). β-amino acid substitution in the SIINFEKL antigen alters immunological recognition. Cancer Biol. Ther..

[30] Rastogi I., Moseman J.E., Jeon D., Muralidhar A., McNeel D.G. (2025). Evaluation of agents that affect anti-tumor function of CD8+ T cells when employed at the time of T-cell activation. Methods in Cell Biology, Immuno-Oncology and Immunotherapy.

[31] van de Laar L., Coffer P.J., Woltman A.M. (2012). Regulation of dendritic cell development by GM-CSF: Molecular control and implications for immune homeostasis and therapy. Blood.

[32] Granato A., Hayashi E.A., Baptista B.J., Bellio M., Nobrega A. (2018). Correction: IL-4 Regulates Bim Expression and Promotes B Cell Maturation in Synergy with BAFF Conferring Resistance to Cell Death at Negative Selection Checkpoints. J. Immunol..

[33] Ahn J.S., Agrawal B. (2005). IL-4 is more effective than IL-13 for in vitro differentiation of dendritic cells from peripheral blood mononuclear cells. Int. Immunol..

[34] Deng J., Pennati A., Cohen J.B., Wu Y., Ng S., Wu J.H., Flowers C.R., Galipeau J. (2016). GIFT4 fusokine converts leukemic B cells into immune helper cells. J. Transl. Med..

[35] Eksioglu E.A., Mahmood S.S., Chang M., Reddy V. (2007). GM-CSF promotes differentiation of human dendritic cells and T lymphocytes toward a predominantly type 1 proinflammatory response. Exp. Hematol..

[36] Shi Y., Liu C.H., Roberts A.I., Das J., Xu G., Ren G., Zhang Y., Zhang L., Yuan Z.R., Tan H.S. (2006). Granulocyte-macrophage colony-stimulating factor (GM-CSF) and T-cell responses: What we do and don’t know. Cell Res..

[37] Kumar A., Khani A.T., Ortiz A.S., Swaminathan S. (2022). GM-CSF: A Double-Edged Sword in Cancer Immunotherapy. Front. Immunol..

[38] Riou C., Dumont A.R., Yassine-Diab B., Haddad E.K., Sekaly R.-P. (2006). IL-4 influences the differentiation and the susceptibility to activation-induced cell death of human naive CD8+ T cells. Int. Immunol..

[39] Chen Q., He F., Kwang J., Chan J.K.Y., Chen J. (2012). GM-CSF and IL-4 Stimulate Antibody Responses in Humanized Mice by Promoting T, B, and Dendritic Cell Maturation. J. Immunol..

[40] Sweet M.J., Hume D.A. (1996). Endotoxin signal transduction in macrophages. J. Leukoc. Biol..

[41] Mosier D., Subbarao B. (1982). Thymus-independent antigens: Complexity of B-lymphocyte activation revealed. Immunol. Today.

[42] Xu H., Liew L.N., Kuo I.C., Huang C.H., Goh D.L., Chua K.Y. (2008). The modulatory effects of lipopolysaccharide-stimulated B cells on differential T-cell polarization. Immunology.

[43] Kleindienst P., Brocker T. (2005). Concerted antigen presentation by dendritic cells and B cells is necessary for optimal CD4 T-cell immunity in vivo. Immunology.

[44] Parekh V.V., Prasad D.V.R., Banerjee P.P., Joshi B.N., Kumar A., Mishra G.C. (2003). B Cells Activated by Lipopolysaccharide, But Not By Anti-Ig and Anti-CD40 Antibody, Induce Anergy in CD8+ T Cells: Role of TGF-β1. J. Immunol..

[45] Wennhold K., Weber T.M., Klein-Gonzalez N., Thelen M., Garcia-Marquez M., Chakupurakal G., Fiedler A., Schlösser H.A., Fischer R., Theurich S. (2016). CD40-activated B cells induce anti-tumor immunity *in vivo*. Oncotarget.

[46] Ibáñez-Vea M., Zuazo M., Gato M., Arasanz H., Fernández-Hinojal G., Escors D., Kochan G. (2017). Myeloid-Derived Suppressor Cells in the Tumor Microenvironment: Current Knowledge and Future Perspectives. Arch. Immunol. Ther. Exp..

[47] Seyfizadeh N., Muthuswamy R., Mitchell D.A., Nierkens S. (2016). Migration of dendritic cells to the lymph nodes and its enhancement to drive anti-tumor responses. Crit. Rev. Oncol..

[48] Sorenmo K.U., Krick E., Coughlin C.M., Overley B., Gregor T.P., Vonderheide R.H., Mason N.J., Bernhard E.J. (2011). CD40-Activated B Cell Cancer Vaccine Improves Second Clinical Remission and Survival in Privately Owned Dogs with Non-Hodgkin’s Lymphoma. PLoS ONE.

[49] Wennhold K., Shimabukuro-Vornhagen A., von Bergwelt-Baildon M. (2019). B Cell-Based Cancer Immunotherapy. Transfus. Med. Hemotherapy.

