# Supplementary material for: CD8+ T Cells Primed by Antigenic Peptide-Pulsed B Cells or Dendritic Cells Generate Similar Anti-Tumor Response
Source: Vaccines (Basel). 2025 Sep 6;13(9):953. doi: 10.3390/vaccines13090953 (PMC12474392; doi:10.3390/vaccines13090953)
Supplement: Supplementary file 1 [file vaccines-13-00953-s001.zip › vaccines-3776707-supplementary.pdf]

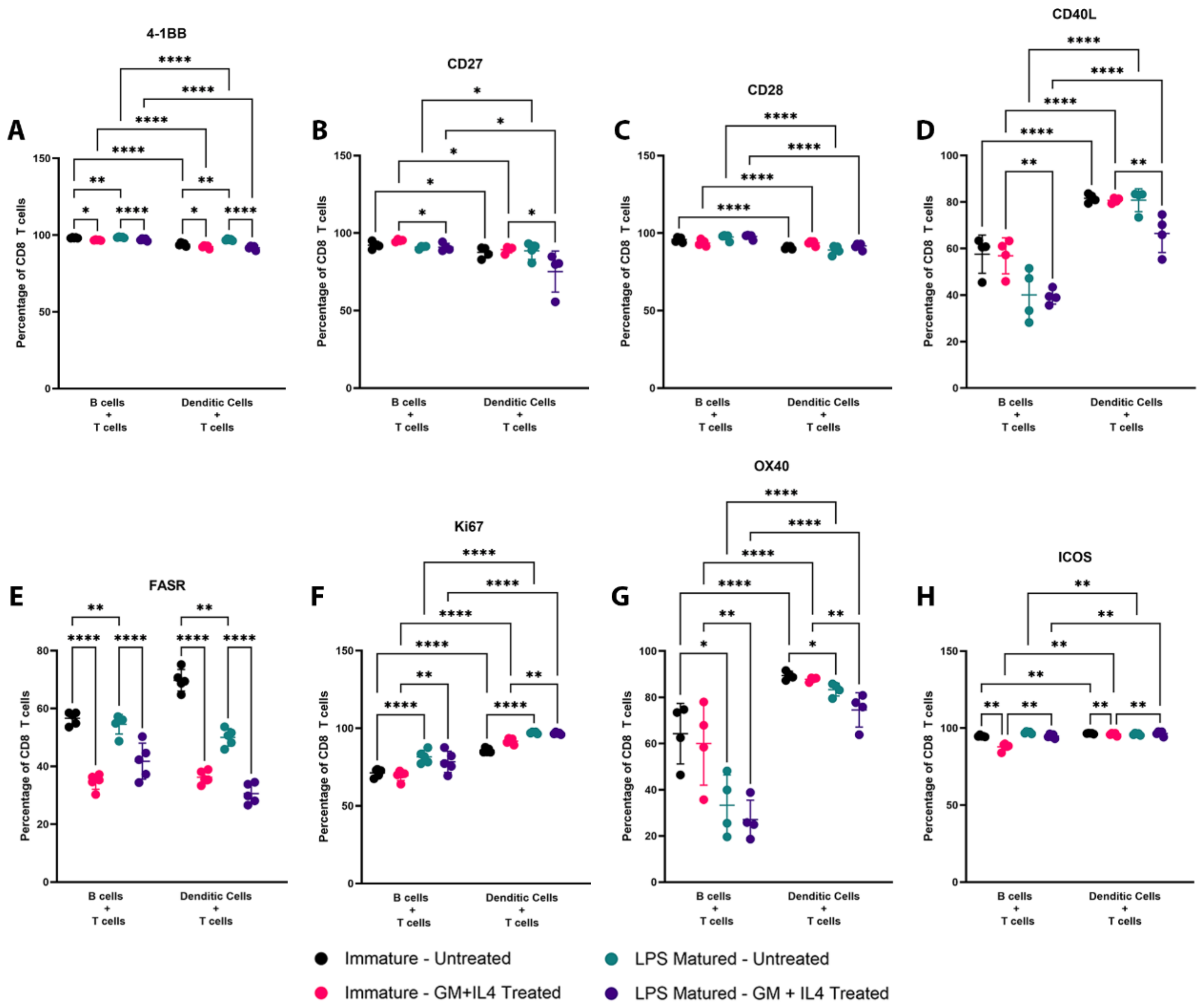

Sup Figure S1: Epitope-specific priming of CD8+ T cells by B cells and DCs resulted in different activation marker expression profiles *In vitro* assay was set-up as in Fig 1B. Flow cytometry was performed to measure the percentage of CD8+ T cells positive for expression of activation markers (A) 4-1BB, (B) CD27, (C) CD28, (D) CD40L, (E) FASR, (F) Ki67, (G) OX40, and (H) ICOS. Each data point represents a biological replicate, with mean and standard deviation from five replicates. Asterisks * indicate $p<0.05$, ** indicate $p<0.01$, and **** indicate $p<0.0001$. Results are from one experiment and are representative of at least two similar, independent experiments.

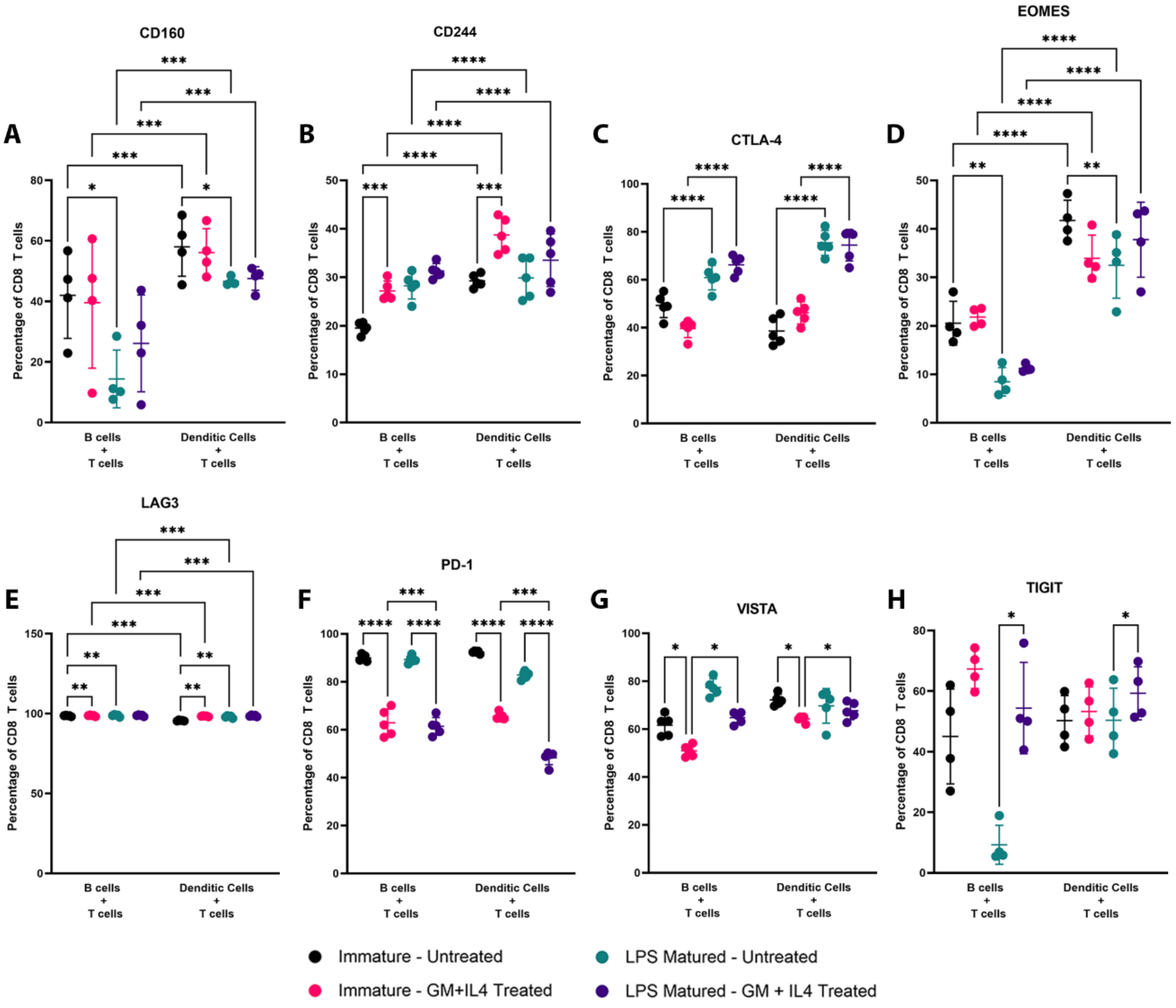

Sup Figure S2: Epitope-specific priming of CD8+ T cells by B cells and DCs resulted in different checkpoint and exhaustion related marker expression profiles.

In vitro assay was set-up as in Fig 1B. Flow cytometry was performed to measure the percentage of CD8+ T cells positive for expression of checkpoint and exhaustion markers (A) CD160, (B) CD244, (C) CTLA-4, (D) EOMES, (E) LAG3, (F) PD1, (G) VISTA, and (H) TIGIT. Each data point represents a biological replicate, with mean and standard deviation from five replicates. Asterisks * indicate p<0.05, ** indicate p<0.01, *** indicate p<0.001, and **** indicate p<0.0001. Results are from one experiment and are representative of at least two similar, independent experiments.

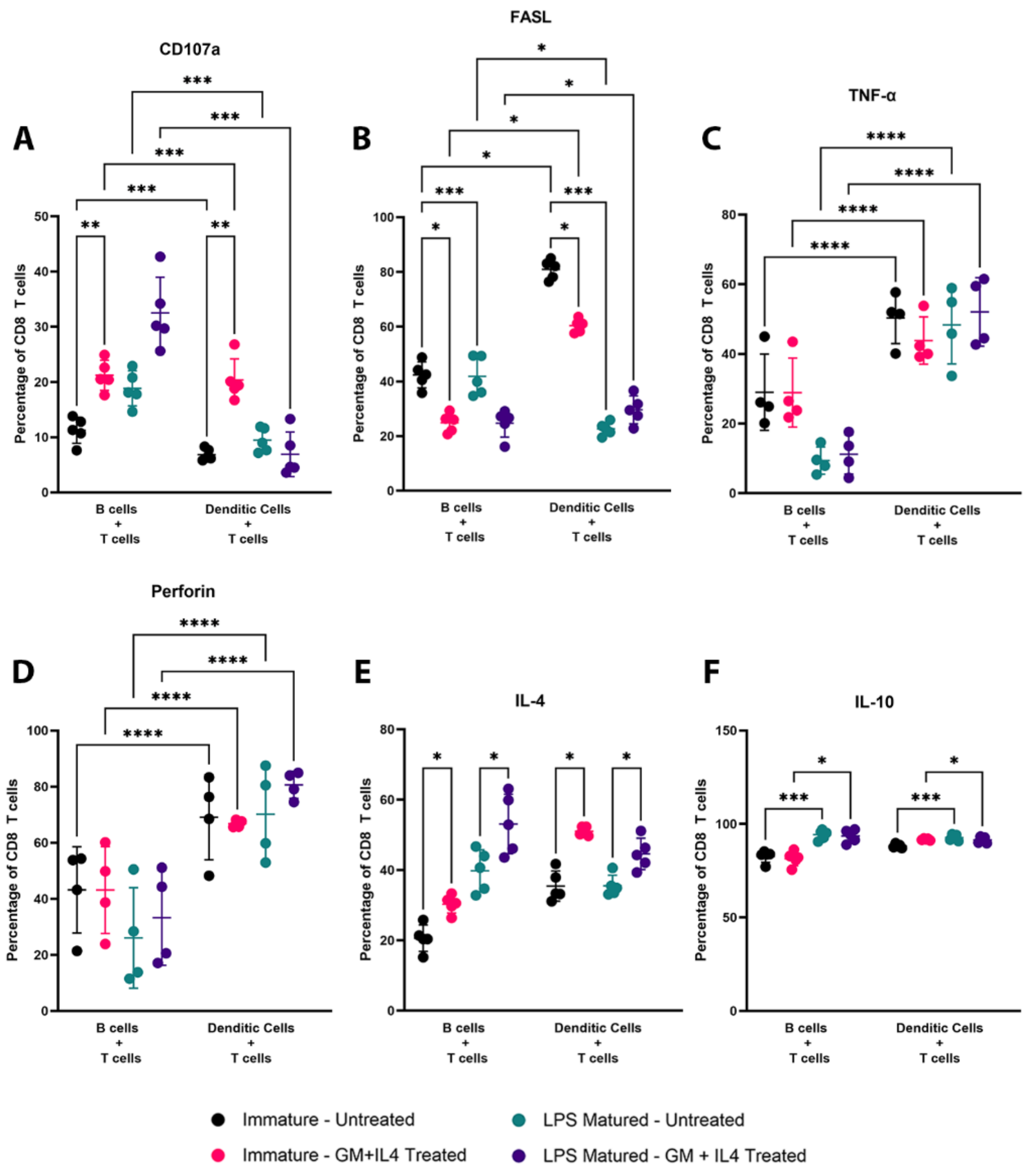

Sup Figure 3S: Priming by B cells and DCs resulted in differences in expression of cytotoxicity related markers, and IL-4 and IL-10, on CD8+ T cells.

In vitro assay was set up as in Fig 1B. Flow cytometry was performed to measure the percentage of CD8+ T cells positive for expression of surface and intra-cellular proteins associated with cytotoxicity. Expression levels of (A) CD107a, (B)FASL, and intraceullar expression levels of (C) TNF-α, (D) Perforin, (E) IL-4 and (F) IL-10 were also quantified. Each data point represents a biological replicate, with mean and standard deviation from five replicates. Asterisks * indicate p<0.05 ** indicate p<0.01, *** indicate p<0.001, and **** indicate p<0.0001. Results are from one experiment, with samples assessed using five biological replicates.

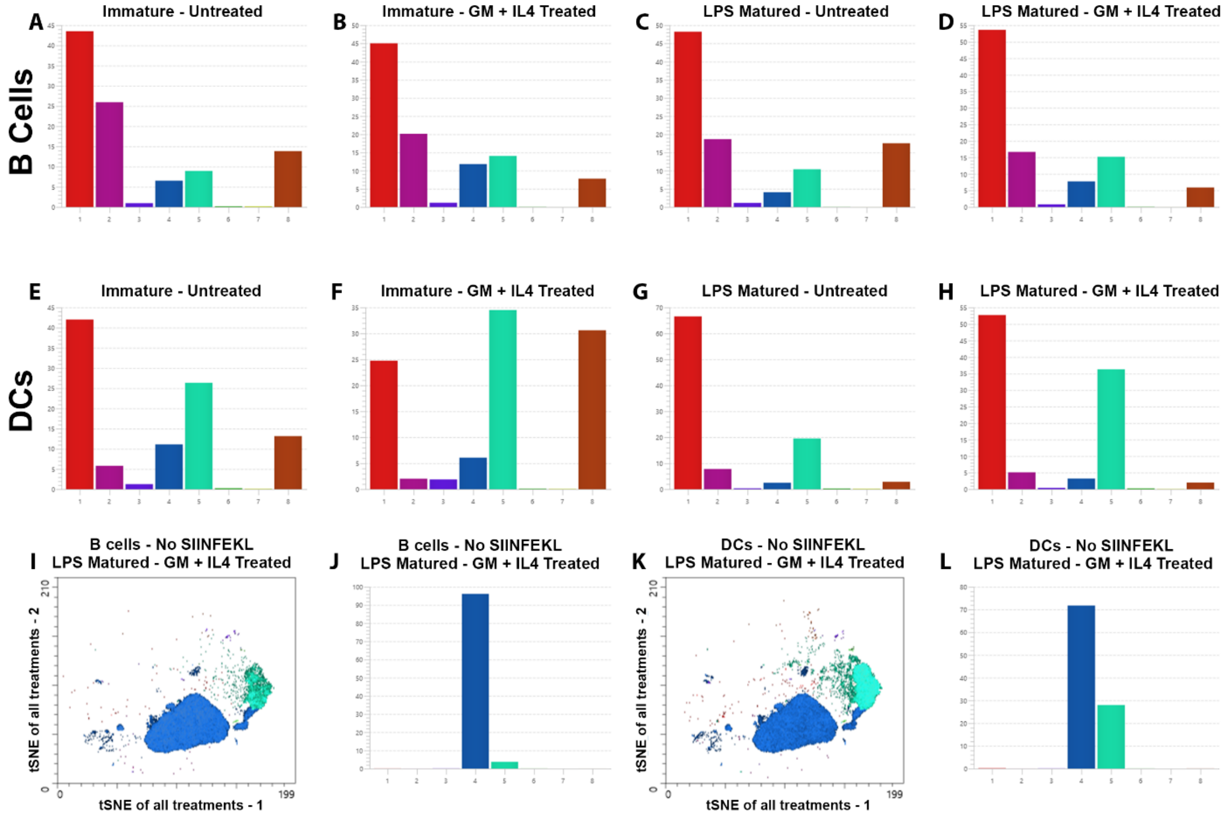

Sup Figure S4: tSNE analysis revealed different phenotypes of CD8+ T cells resulting from priming by B cells and DCs.

In vitro assay was set up as in Fig 1B. Flow cytometry was performed to measure expression of markers used in the analysis. tSNE analysis was performed using FlowJo (version 10.10), based on expression levels of 4-1BB, EOMES, IFN-γ, IL-10, IL-4, Ki67, Perforin and TNF-α. (A-H, J and L) Percentage of clusters identified from the tSNE analysis are presented as bar graphs for all treatment groups and (I, K) individual tSNE plots for non-primed groups are presented. Data represented is from one experiment with five biological replicates for each treatment group.

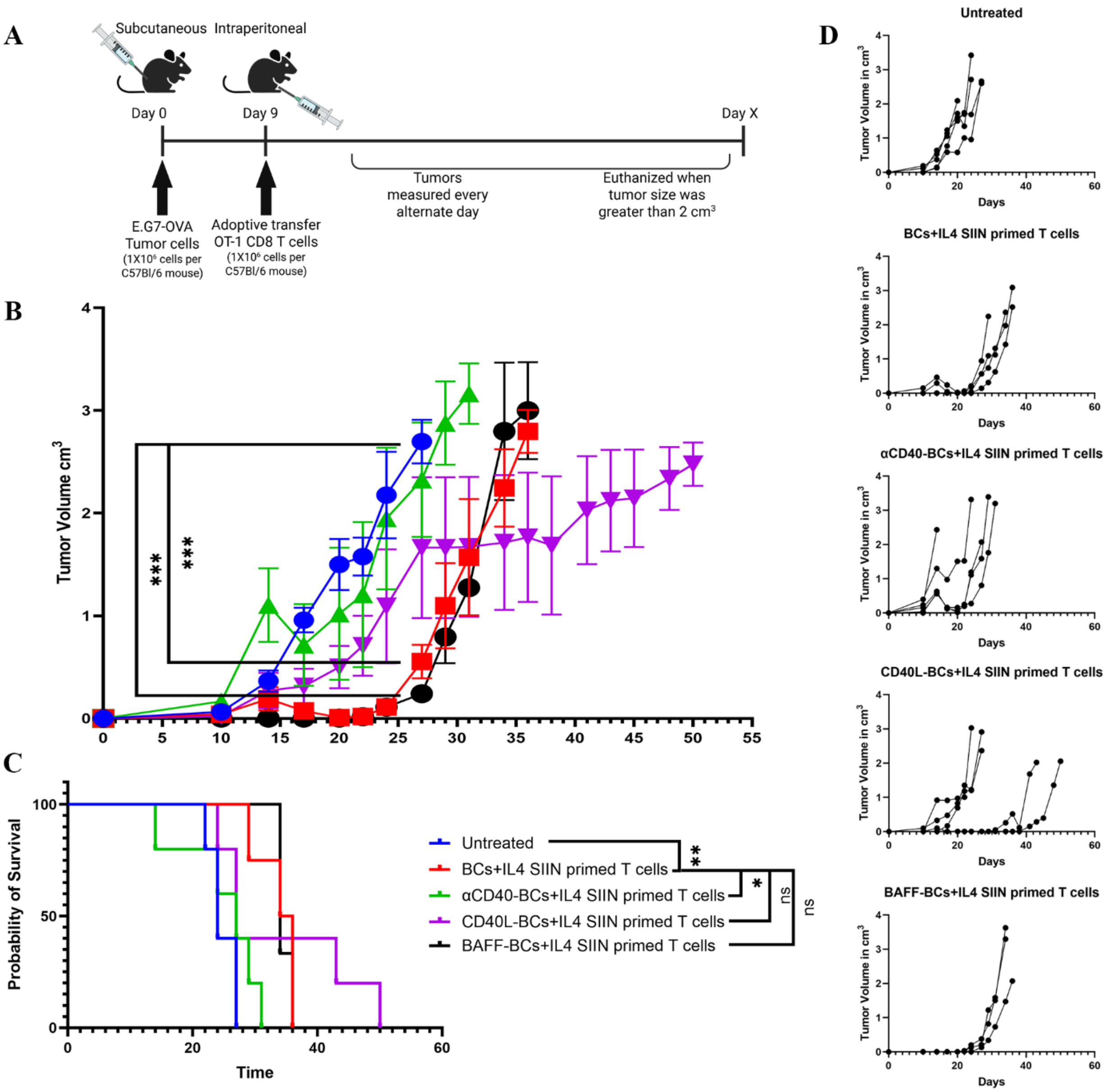

Sup Figure S5: CD8+ T cells primed by matured B cells did not improve anti-tumor response. C57Bl/6 mice were implanted with E.G7-OVA tumors subcutaneously, with three to six mice per treatment group. CD8+ T cells were primed *in vitro* as in Fig 1B by matured-B cells in the presence of IL-4, and after 48 hours of incubation CD8+ T cells were re-purified using negative selection. 10^6 re-purified CD8+ T cells were adoptively transferred to individual tumor-bearing mice on Day 9, via intra-peritoneal injection. (A) Schematic and timeline. (B) Tumor volumes (cm^3) plotted against the day of measurement, (C) survival curve evaluating time to death or tumor volume $\geq$ 2 cm^3, whichever occurred first and (D) individual tumor curves for each treatment group are presented. Asterisks * indicate p<0.05 and ** indicate p<0.01. Results presented are from one experiment, but data are representative of at least two similar, independent experiments.

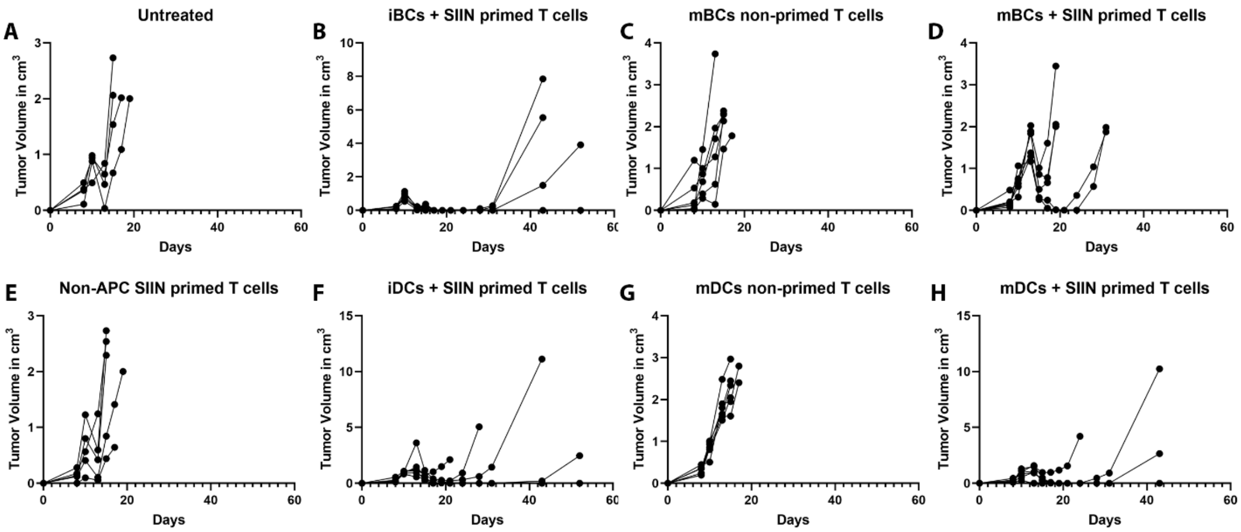

Sup Figure S6: CD8+ T cells primed by immature B cells, mature DCs, and immature DCs generated similar anti-tumor response.

C57Bl/6 mice were implanted with E.G7-OVA tumors subcutaneously, with six mice per treatment group (n=4 for negative control groups). CD8+ T cells were primed *in vitro* as in Fig 1B in the presence of GM-CSF and IL-4, and after 48 hours of incubation CD8+ T cells were re-purified using negative selection. 10^6 re-purified CD8+ T cells were adoptively transferred to individual tumor-bearing mice on Day 9, via intra-peritoneal injection. (A-H) Tumor volumes (cm^3) for individual treatment groups are plotted against the day of measurement until time to death or tumor volume $\geq$ 2 cm^3, whichever occurred first. Results presented are from one experiment, but data are representative of at least two similar, independent experiments.
